# Anomalous origin of the left coronary artery from the pulmonary artery with coronary artery fistula in an adult presenting with cardiomegaly, abnormal electrocardiogram, myocardial perfusion defect and pulmonary hypertension: a case report

**DOI:** 10.3389/fcvm.2026.1844272

**Published:** 2026-08-19

**Authors:** Lian Luo, Xuefeng Wang, Kerui Huang, Yutong Yang, Bo Zhu, Gang Wei

**Affiliations:** 1 Department of Cardiovascular Medicine, 363 Hospital, Chengdu, Sichuan, China; 2 Department of Cardiology, The Affiliated Hospital, Southwest Medical University, Luzhou, Sichuan, China

**Keywords:** adult, ALCAPA, case report, myocardial ischemia, pulmonary hypertension

## Abstract

The anomalous origin of the left coronary artery from the pulmonary artery (ALCAPA) represents a rare and potentially life-threatening coronary anomaly. The clinical manifestations, progression, and prognosis of ALCAPA are significantly influenced by the degree of collateral circulation. Only 10%–15% of untreated individuals with ALCAPA develop extensive collateral circulation from the right coronary artery, enabling them to survive into adulthood with either no symptoms or various nonspecific symptoms, thereby presenting considerable diagnostic challenges. Furthermore, ALCAPA may occur in isolation or in conjunction with other congenital heart diseases, which can obscure its presence and complicate its management. Herein, we present a distinctive case of an adult patient with ALCAPA and coronary-pulmonary artery fistula, who exhibited facial edema and mild pericardial effusion secondary to severe hypothyroidism, without any cardiac symptoms. Given the patient's cardiomegaly, coronary artery calcification, abnormal electrocardiogram, and hyperlipidemia, coronary angiography was conducted, which incidentally revealed the presence of ALCAPA and the coronary-pulmonary artery fistula. Subsequent right heart catheterization and resting single-photon emission computed tomography myocardial perfusion scintigraphy identified pre-capillary pulmonary hypertension and regional myocardial perfusion defect in the anterior and lateral walls of the left ventricle. This case underscores the considerable difficulty in diagnosing adult ALCAPA and emphasizes the need for a thorough evaluation in patients presenting with atypical cardiac manifestations.

## Introduction

The anomalous origin of the left coronary artery from the pulmonary artery (ALCAPA) represents a rare congenital coronary anomaly, with an estimated prevalence of approximately 1 in 300,000 live births (1). The clinical manifestations, progression, and prognosis of ALCAPA are significantly influenced by the degree of collateral circulation (2). If left untreated, more than 90% of individuals with ALCAPA succumb to severe heart failure during infancy due to inadequate formation of collateral circulation (3). Conversely, 10%–15% of patients may develop extensive collateral circulation originating from the right coronary artery, allowing them to survive into adulthood with either no symptoms or a range of nonspecific symptoms, thereby presenting considerable diagnostic challenges (4). In such cases, the entire left ventricle is perfused by the low-pressure collateral circulation, with the majority of blood flow directed into the main pulmonary artery rather than the myocardial capillaries. This can lead to silent myocardial ischemia, myocardial scarring, cardiac dysfunction, ventricular arrhythmia, and even sudden cardiac death (2). Furthermore, ALCAPA can present either in isolation or alongside other congenital heart anomalies, such as atrial septal defect, ventricular septal defect, aortic coarctation, tetralogy of Fallot, bicuspid aortic valve, and coronary artery fistula, which can obscure its presence and complicate its clinical management (5, 6).

The case we present underscores the considerable diagnostic challenges encountered in adult patients and emphasizes the importance of thorough evaluation in individuals exhibiting atypical cardiac manifestations. A high degree of clinical suspicion is essential when faced with unexplained cardiomegaly, pulmonary artery dilation, mitral regurgitation, and/or abnormal electrocardiogram findings, even in young and/or asymptomatic patients (3). Furthermore, the diagnosis of ALCAPA often necessitates a multimodal approach, incorporating electrocardiography, echocardiography, coronary computed tomography angiography, cardiac magnetic resonance imaging, coronary angiography, and myocardial perfusion scintigraphy, to accurately characterized the anomalous coronary anatomy and its associated hemodynamic implications (1, 7).

## Case presentation

A 55-year-old female farmer was referred to our center presenting with a one-week history of facial edema. The patient reported no cardiorespiratory symptoms, including chest tightness, chest pain, dyspnea, syncope, cough, sputum production, or lower limb edema. Her medical history was notable for renal stones a decade earlier, with no reported family history of sudden cardiac death or hereditary disease.

## Diagnostic assessment and therapeutic intervention

Upon physical examination, the patient's body mass index was calculated at 28.9 Kg/m^2^, and the blood pressure was measured at 117/60 mm Hg, with a heart rate of 68 beats per minute. The patient's general condition was stable, although cardiomegaly was observed without significant abnormalities on cardiopulmonary auscultation.

Laboratory investigations, including complete blood cell counts, renal and liver function tests, electrolyte levels, coagulation profile, NT-pro BNP, and high-sensitivity troponin I levels, were largely within normal limits. However, thyroid function tests indicated severe hypothyroidism, as demonstrated by free triiodothyronine (FT3) levels of <0.7 pg/mL (reference range: 1.8–3.8 pg/mL), free thyroxine (FT4) levels of <0.10 ng/dL (reference range: 0.78–1.86 ng/dL), and thyroid-stimulating hormone (TSH) levels of >100.00 mIU/L (reference range: 0.38–5.57 mIU/L). Additionally, thyroglobulin antibody (TgAb) levels were >4000.00 IU/mL (reference range: 0.00–120.00 IU/mL), and thyroid peroxidase antibody (TPOAb) levels were 349.39 IU/mL (reference range: 0.00–35.00 IU/mL). Furthermore, the lipid profile indicated mixed hyperlipidemia, with triglycerides measured at 3.54 mmol/L (reference range: 0.40–1.70 mmol/L), high-density lipoprotein cholesterol at 1.63 mmol/L (reference range: 1.04–1.55 mmol/L), and low-density lipoprotein cholesterol at 4.45 mmol/L (reference range: 1.00–3.37 mmol/L).

**Figure 1:**
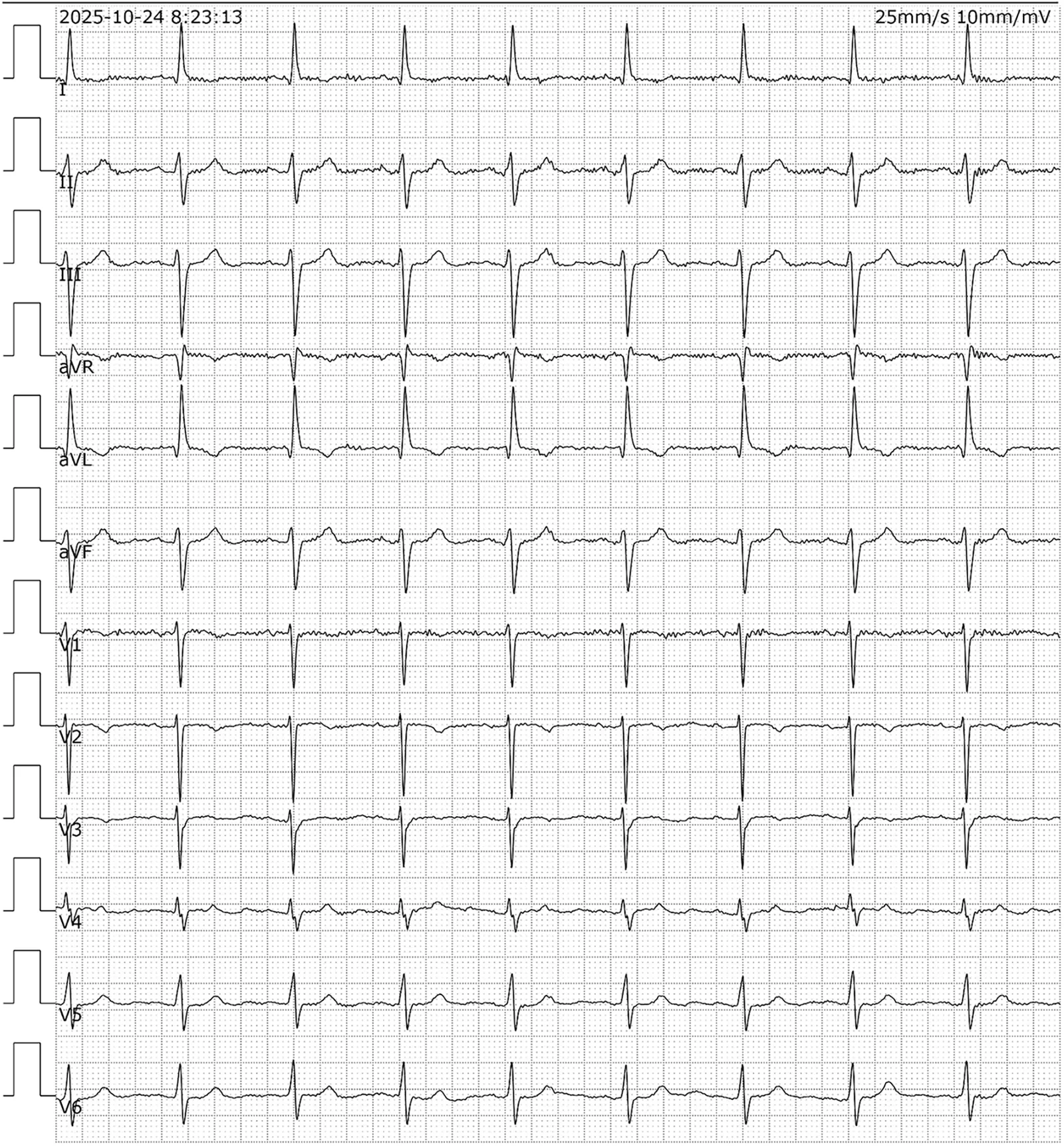
Electrocardiogram showing sinus rhythm, left anterior fascicular block, and clockwise rotation.

A non-contrast chest computed tomography conducted at the previous hospital identified cardiomegaly, pulmonary artery dilation, mild pericardial effusion, and moderate coronary artery calcification. An electrocardiogram revealed sinus rhythm, left anterior fascicular block, and clockwise rotation (Figure 1). Transthoracic echocardiography indicated biatrial dilation (left atrial diameter: 38 mm; right atrial diameter: 51 mm), mild aortic and mitral regurgitation, and a preserved left ventricular ejection fraction (LVEF: 70%).

**Figure 2:**
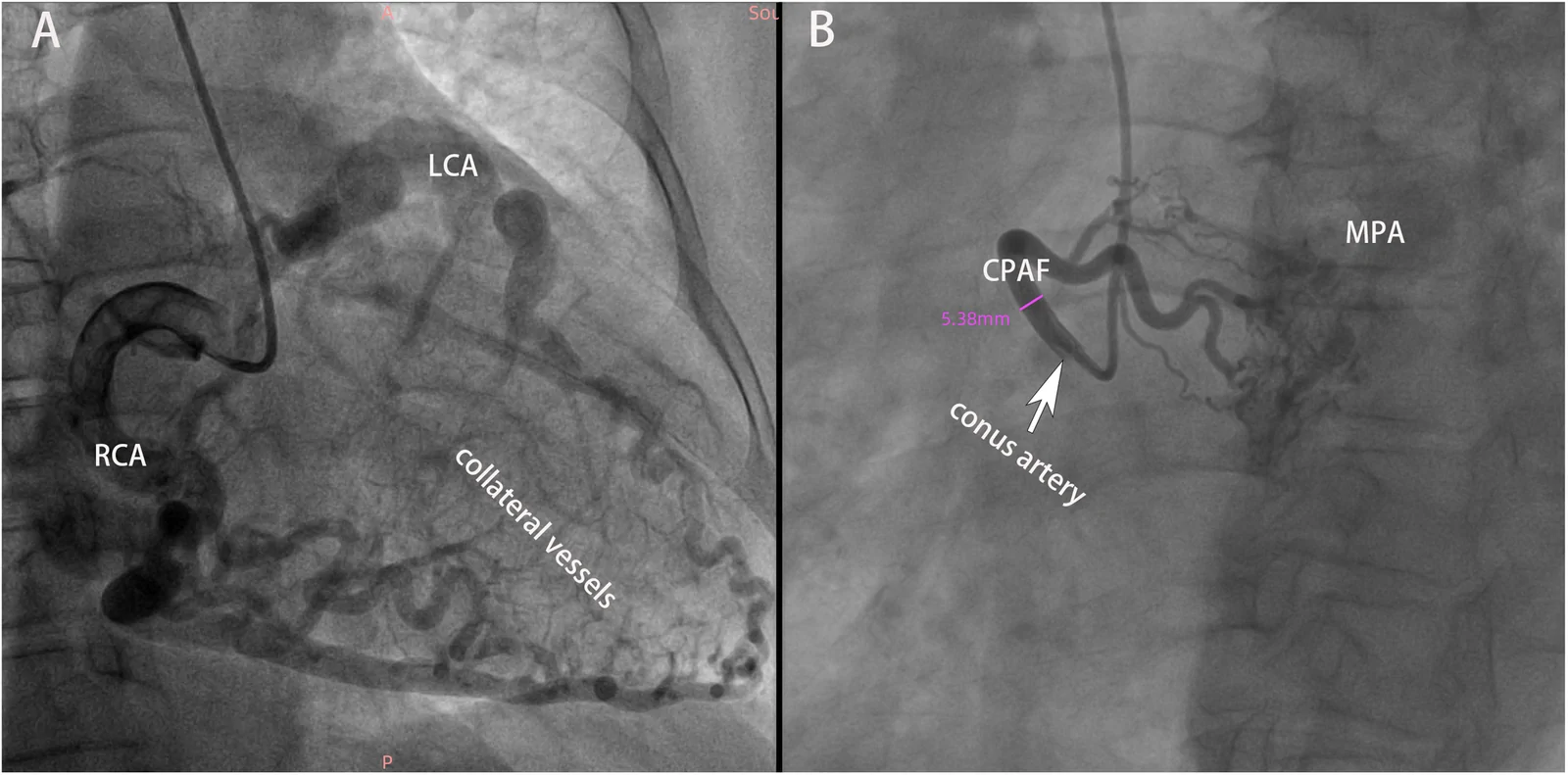
Right coronary angiogram showing an anomalous origin of the left coronary artery from the pulmonary artery **(A)** and a coronary-pulmonary artery fistula **(B)**.

**Figure 3:**
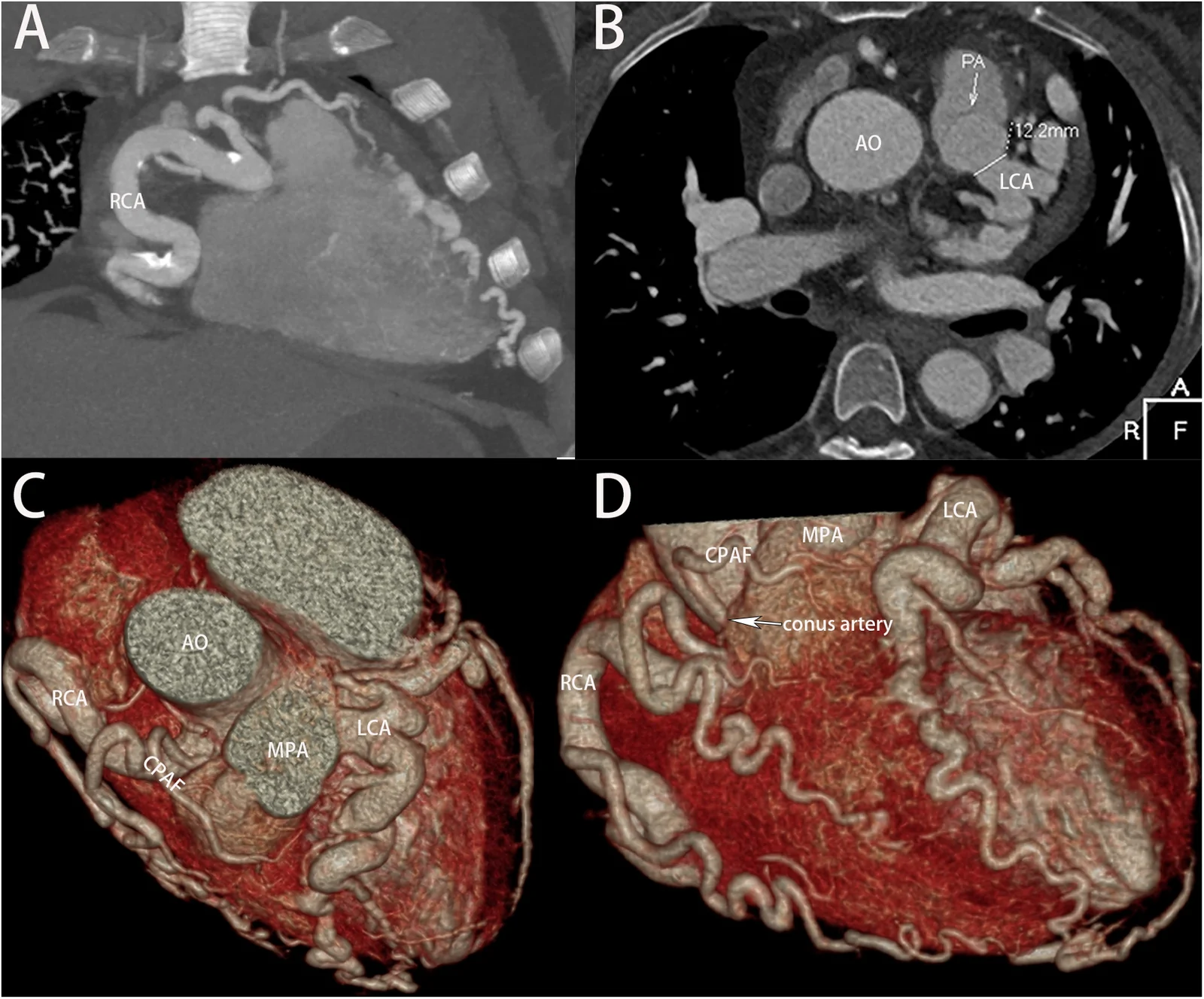
Coronary computed tomography angiogram showing right coronary artery calcification **(A)**, an enlarged left coronary artery **(B)**, an anomalous left coronary artery and a coronary-pulmonary artery fistula **(C,D)**.

**Figure 4:**
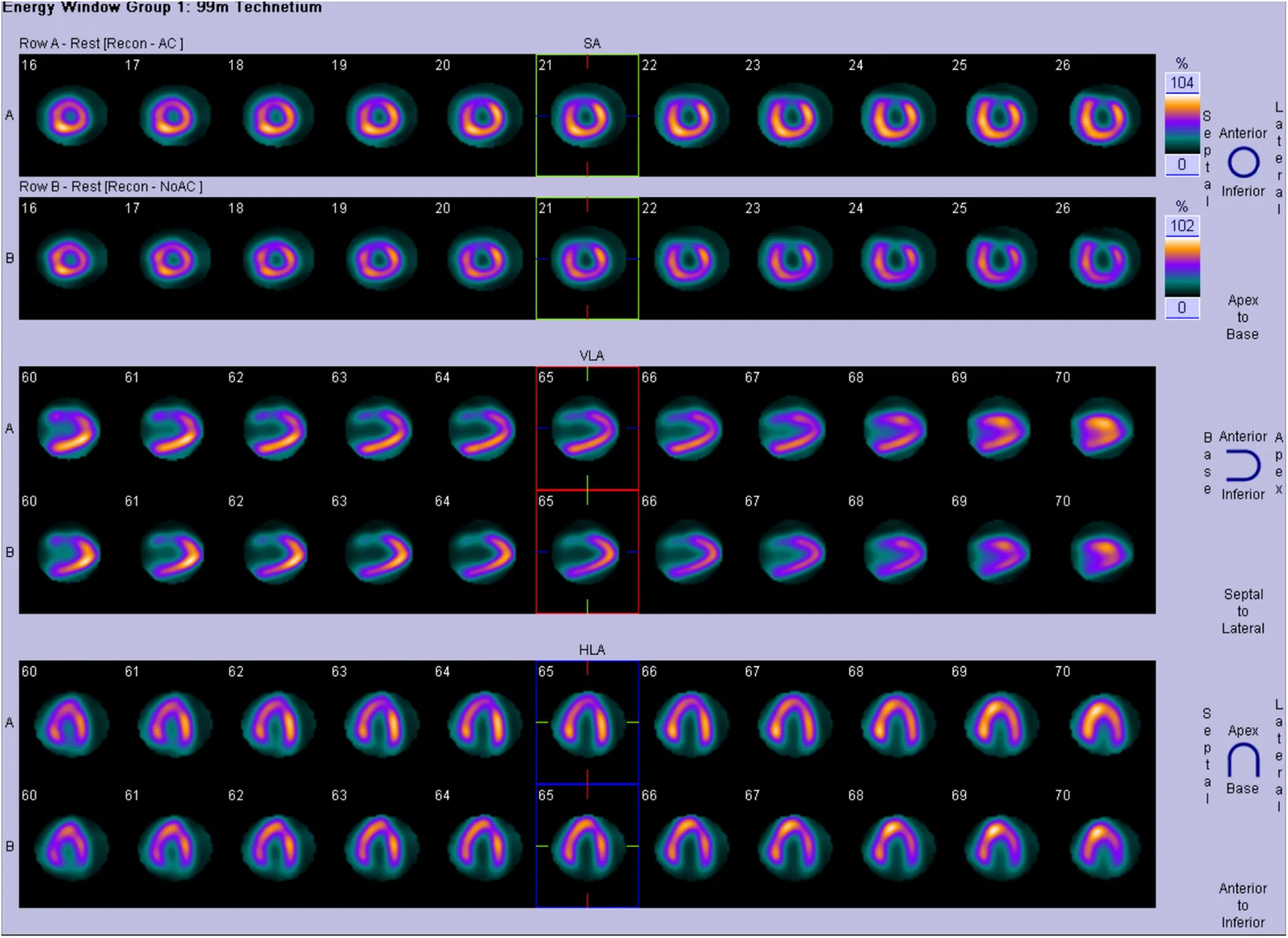
Myocardial perfusion scintigraphy at rest showing significant perfusion defects in the anterior and lateral walls of the left ventricle.

Given the presence of cardiomegaly, moderate coronary artery calcification, an abnormal electrocardiogram, and hyperlipidemia, coronary angiography was undertaken to rule out coronary artery disease. During the angiographic procedure, the catheter was unable to intubate the left coronary artery (LCA), and subsequent left coronary sinus angiography revealed the absence of the left coronary artery. Notably, right coronary angiography revealed a markedly enlarged right coronary artery (RCA) that originated from the right coronary sinus, subsequently filling extensive collateral vessels and the LCA system in a retrograde manner, and finally draining into the main pulmonary trunk (Figure 2A; Supplementary Video S1). Additionally, a coronary artery fistula (CAF) originating from the conus artery and draining into the main pulmonary artery trunk was identified (Figure 2B; Supplementary Video S2). Subsequent coronary computed tomography angiography corroborated the diagnosis of an anomalous origin of the left coronary artery from the pulmonary artery (ALCAPA) accompanied by a coronary-pulmonary artery fistula (CPAF), and revealed calcification in the RCA (Figure 3). Further evaluation through right heart catheterization revealed pre-capillary pulmonary hypertension, characterized by a mean pulmonary artery pressure of 22 mmHg, a pulmonary artery wedge pressure of 10 mmHg, a pulmonary vascular resistance of 2.4 Wood units, and a Qp:Qs ratio of 1.6 (Supplementary Table S1). Single-photon emission computed tomography myocardial perfusion scintigraphy at rest demonstrated significant perfusion defects in the anterior and lateral walls of the left ventricle (Figure 4).

Considering the patient's significant myocardial perfusion defects, pre-capillary pulmonary hypertension, biatrial dilation, and valvular regurgitation, there exists a considerable risk of progressing to cardiac dysfunction and sudden cardiac death. Following a multidisciplinary heart team discussion, surgical intervention to correct the ALCAPA and close the CPAF was planned, contingent upon the normalization of thyroid function. The patient received explicit counseling regarding the elevated risk of sudden cardiac death and heart failure. Nevertheless, the patient opted against surgical intervention, citing personal preferences and apprehensions about the associated surgical risks. Consequently, thyroid hormone replacement therapy was initiated with levothyroxine, starting at a dose of 12.5 µg once daily and gradually titrated to 62.5 µg once daily. Concurrently, lipid-lowering treatment with atorvastatin at a dose of 20 mg nightly was commenced. The patient was also advised to refrain from engaging in vigorous physical activities. Adherence to the prescribed medication regimen was satisfactory, leading to the resolution of facial edema and the normalization of thyroid function within one month, as well as the normalization of the lipid profile within three months. However, a follow-up echocardiogram conducted three months later revealed no improvement in biatrial dilation, with mild aortic and mitral regurgitation persisting. Over a five-month follow-up period, the patient remained asymptomatic and in good health. It was recommended that the patient undergo regular clinical follow-ups every 3–6 months, along with annual echocardiograms, myocardial perfusion scintigraphy, and ambulatory electrocardiograms, to monitor for disease progression or arrhythmias.

## Discussion

The anomalous origin of the left coronary artery from the pulmonary artery (ALCAPA), also known as Bland-White-Garland syndrome, is a rare and potentially life-threatening congenital coronary anomaly. It occurs in approximately 1 in 300,000 live births and constitutes 0.25%–0.5% of all congenital heart diseases (1). The clinical manifestations, progression, and prognosis of ALCAPA are significantly influenced by the degree of collateral circulation (2). If untreated, over 90% of ALCAPA patients do not develop adequate collateral circulation and succumb to severe heart failure during infancy (8). Conversely, 10%–15% of patients may develop extensive collateral circulation from the RCA and survive into adulthood, exhibiting either no symptoms or various nonspecific symptoms (4). Nevertheless, the perfusion of LCA is markedly impaired due to the fact that the entire left ventricle relies on low-pressure collateral circulation (8). Furthermore, a substantial portion of the blood is diverted into the lower-resistance pulmonary artery instead of the higher-resistance myocardial capillaries (8). This redistribution results in a “coronary steal” phenomenon (2). Consequently, silent myocardial ischemia occurs, placing patients with ALCAPA at risk for myocardial scarring, cardiac remodeling and dysfunction, valve regurgitation, ventricular arrhythmias, and even sudden cardiac death (5, 9).

Without surgical intervention, 80% to 90% of these adult ALCAPA patients succumb to sudden death at a median age of 35 years (7). Additionally, 62% of patients presenting with life-threatening conditions exhibit no prior symptoms (10). While the natural progression of the disease results in a high incidence of sudden death in early adulthood (under 50 years), the risk of sudden death appears to decline after the age of 50 (2% compared to 9%), despite a lower frequency of surgical interventions in this age group (63% compared to 96%) (4, 10). Currently, there is a paucity of high-quality evidence to guide management strategies for asymptomatic older adult patients with ALCAPA. In this context, a comprehensive evaluation of the risk of sudden cardiac death, employing echocardiography, ambulatory electrocardiogram, treadmill testing, resting/stress myocardial perfusion scintigraphy, and cardiac magnetic resonance imaging, may improve the risk stratification of asymptomatic older adult patients with ALCAPA. In our patient, the extensive collateral vessels originating from the enlarged RCA have been crucial for her prolonged well-being. However, her resting myocardial perfusion defect in the LCA territory would probably exacerbate with exertion and have long-term clinical implications. Therefore, timely surgical intervention is essential to salvage the endangered myocardium and prevent fatal complications. Unfortunately, she declined to undergo cardiac surgery. In this context, it is crucial to implement exercise restrictions and maintain close monitoring for ischemic symptoms, disease progression and arrhythmias.

In adult-type ALCAPA, patients often present with either no symptoms or a range of nonspecific symptoms, including angina, dyspnea, ventricular arrhythmia, and even sudden cardiac death (4, 7, 11). This variability poses significant diagnostic challenges, especially in asymptomatic and/or young adult patients. Furthermore, ALCAPA may occur in isolation or in conjunction with other congenital heart anomalies, such as atrial septal defect, ventricular septal defect, aortic coarctation, tetralogy of Fallot, bicuspid aortic valve, and coronary artery fistula (CAF) (5, 6). These coexisting conditions can obscure the presence of ALCAPA and complicate its clinical management. In practice, ALCAPA is frequently misdiagnosed as other conditions, such as dilated cardiomyopathy, endocardial fibroelastosis, CAF, isolated mitral valve lesions, or other isolated congenital heart disease (1, 3, 5). In our case, the patient exhibited no cardiac symptoms; however, the presence of cardiomegaly, coronary artery calcification, abnormal electrocardiogram, and hyperlipidemia prompted further investigation into potential underlying causes. Cardiac dilation, pericardial effusion, abnormal electrocardiogram, and hyperlipidemia are recognized complications of hypothyroidism (12). Furthermore, hypothyroidism may exacerbate the hemodynamic burden associated with ALCAPA and CAF. Considering that a three-month course of thyroid hormone replacement therapy may be insufficient for achieving complete reversal of cardiovascular remodeling following prolonged severe hypothyroidism, the cardiomegaly, pulmonary artery dilation, biatrial dilation, and valvular regurgitation observed in our patient may be attributed to ALCAPA, CAF, and concurrent hypothyroidism. Additionally, the calcification of the patient's RCA calcification may be secondary to aging and/or hyperlipidemia. Furthermore, the left anterior fascicular block may result from prolonged ischemic insult, as previously documented in our earlier case of ALCAPA (2). Finally, the clockwise rotation observed on the electrocardiogram can be attributed to the reduced voltage of the left ventricle, resulting from myocardial ischemia/fibrosis and/or severe hypothyroidism, which fails to counterbalance the electrocardiac vector of the right ventricle. Consequently, a heightened level of clinical suspicion is necessary when confronted with unexplained cardiomegaly, pulmonary artery dilation, mitral regurgitation, ischemic changes on the electrocardiogram, left anterior fascicular block, or clockwise rotation, even in young and/or asymptomatic individuals (1, 4, 13).

The diagnosis of ALCAPA often necessitates a multimodal approach, incorporating electrocardiography, echocardiography, coronary computed tomography angiography, cardiac magnetic resonance imaging, coronary angiography and myocardial perfusion scintigraphy to accurately characterize the anomalous anatomy and its associated hemodynamic implications (1, 11, 14). The electrocardiography is a widely accessible and cost-effective diagnostic tool that can provide valuable insights for the diagnosis of ALCAPA. Common electrocardiogram findings in ALCAPA patients include pathological Q waves, ST-T segment changes, and left axis deviation, which should prompt consideration of ALCAPA (1, 4). In clinical settings, echocardiography remains the preferred imaging technique for the rapid assessment of cardiac structure and function. The presence of retrograde blood flow in the LCA and an enlarged RCA are strong indicators of ALCAPA (1). Additional echocardiographic markers include the anomalous origin of the left coronary artery, collateral circulation, abnormal pulmonary artery flow, mitral regurgitation, endocardial fibroelastosis, and left ventricular enlargement and dysfunction (15, 16). Echocardiography is also the preferred modality for postoperative follow-up. Coronary computed tomography angiography offers a noninvasive means to delineate the origin, course, and termination of coronary arteries and adjacent structures, owing to its excellent spatial and temporal resolution (17). It is also regarded as the diagnostic imaging modality of choice for planning surgical interventions (3). In addition to assessing cardiac structure and function, cardiac magnetic resonance imaging offers unique tissue characterization capabilities and can evaluate myocardial ischemia and fibrosis *in vivo* (18). However, invasive coronary angiography continues to be the gold standard diagnostic imaging modality due to its superior resolution (1).

In this intriguing case, our patient exhibited mild pre-capillary pulmonary hypertension in conjunction with a moderate left-to-right shunt, a rare yet frequently overlooked complication associated with ALCAPA and CAF (19, 20). Due to the absence of right heart catheterization in the majority of ALCAPA cases, the actual prevalence and classification of pulmonary hypertension—specifically, whether it is pre-capillary, isolated post-capillary, or combined post- and pre-capillary—remained undetermined. Unlike previous report (19, 21), our patient exhibited pre-capillary pulmonary hypertension. The pathophysiological mechanisms underlying pulmonary hypertension in our patient may be attributed to the volume and pressure overload resulting from both ALCAPA and CPAF, rather than left ventricular dysfunction. In ALCAPA, the significantly enlarged RCA receives substantial blood flow to perfuse the left ventricle via numerous collateral vessels. These blood predominantly drains into the low-resistance main pulmonary artery rather than the high-resistance capillary network, leading to volume and pressure overload and the “coronary steal” phenomenon (2). During coronary angiography, it was observed that a considerable amount of contrast medium filled the enlarged LCA retrogradely through extensive collateral vessels, rapidly draining into the main pulmonary trunk. Similarly, in CPAF, blood from the high-pressure system directly drains into the pulmonary artery, bypassing the high-resistance myocardial capillaries, which also results in volume and pressure overload and the “coronary steal” phenomenon (22). Notably, the concurrence of ALCAPA and CAF is infrequently documented in the literature (6, 23), and this combination has the potential to exacerbate hemodynamic instability. In our patient, echocardiographic evaluation did not reveal elevated pulmonary artery systolic pressure, nor were there other clinical indicators of pulmonary hypertension, aside from cardiomegaly and pulmonary artery dilation (Supplementary Figure S1). However, right heart catheterization indicated the presence of mild pulmonary hypertension. In contrast, Hao's case report of ALCAPA with a coronary-right atrial fistula noted mild right ventricular enlargement, but did not include right heart catheterization (6). In our study, right heart catheterization provided a more comprehensive understanding of the hemodynamic implications associated with the coexistence of ALCAPA and CAF. Additionally, in comparison to the CPAF, the LCA in this patient is substantially larger, thus playing a primary role in the pathogenesis of pulmonary hypertension. In this scenario, surgical intervention to correct the ALCAPA and simultaneously close the CPAF is warranted.

Surgical myocardial revascularization aimed at restoring dual-coronary supply is considered the sole definitive treatment for ALCAPA, even in asymptomatic individuals, due to the persistent risk of ischemia, ventricular arrhythmias, and sudden cardiac death throughout life (4). In the majority of cases, surgical intervention yields favorable outcomes (9, 24, 25). Presently, surgical approaches for ALCAPA primarily encompass direct reimplantation of the anomalous LCA into the ascending aorta, Takeuchi procedure (create an aortopulmonary window and an intrapulmonary tunnel or baffle), and ligation of the LCA accompanied by coronary artery bypass grafting utilizing a saphenous vein graft or left internal mammary artery (4). The practice of isolated LCA ligation has been discontinued due to suboptimal results (3). The coronary reimplantation technique remains the preferred treatment for ALCAPA, especially in infants, owing to their limited collateral circulation and the reduced distance between the LCA ostium and the ascending aorta (3, 24). However, for patients presenting with challenging coronary anatomy, such as a short or tortuous LCA, extensive collateral circulation, or noncompliant tissues surrounding the ALCAPA ostium, the Takeuchi procedure or LCA ligation with coronary artery bypass grafting may be more suitable alternatives (4, 8). Additionally, in adolescent and adult patients with ALCAPA, there is a higher incidence of major adverse cardiovascular events following reimplantation or coronary artery bypass grafting compared to the Takeuchi repair (24). Nonetheless, Takeuchi repair is associated with late complications, including baffle obstruction or leakage, supravalvular pulmonary stenosis, coronary artery aneurysm, intraluminal thrombi, and reintervention (26). In the case of our patient, the LCA is notably enlarged (LCA diameter: 12.2 mm), short, tortuous, and positioned at a considerable distance from the aorta (Figures 3B–D), rendering direct reimplantation impractical. Consequently, the Takeuchi repair emerges as a more suitable option.

## Limitations

This study has several limitations. Firstly, the present study is constrained by the typical limitations associated with a single case analysis of ALCAPA with CPAF. Consequently, the findings should be interpreted with caution. Secondly, the follow-up duration of three months is relatively brief and insufficient to definitively ascertain whether cardiac enlargement, valvular regurgitation, pulmonary artery dilation, and pulmonary hypertension were solely attributable to ALCAPA and CAF. Furthermore, the absence of stress perfusion imaging and cardiac magnetic resonance imaging precludes the assessment of myocardial activity. Lastly, the patient remains at risk for sudden cardiac death and heart failure, yet she declined surgical intervention. In this context, the optimal strategy for follow-up and risk assessment for this patient remains indeterminate.

## Conclusions

In conclusion, this case report delineates an exceedingly rare presentation of ALCAPA in conjunction with a CPAF, incidentally diagnosed in a patient in their sixth decade, presenting with cardiomegaly, an abnormal electrocardiogram, myocardial reperfusion defect, and pulmonary hypertension during an evaluation for severe hypothyroidism. Right heart catheterization provided a more comprehensive understanding of the hemodynamic significance of this coronary anomaly. This case underscores the formidable challenge of diagnosing ALCAPA in adults and emphasizes the critical importance of a thorough and comprehensive assessment in patients exhibiting atypical cardiac manifestations.

## Patient perspective

From the patient's point of view, the therapy was accepted. During a five-month follow-up period, the patient remained asymptomatic and reported an overall sense of well-being.

## Data Availability

The original contributions presented in the study are included in the article/Supplementary Material, further inquiries can be directed to the corresponding authors.

## Supplementary material

Supplementary Figure S1 Computed tomography showing cardiomegaly **(A)**, and pulmonary artery dilation **(B)**.
